# Construction of enhanced MRI-based radiomics models using machine learning algorithms for non-invasive prediction of IL7R expression in high-grade gliomas and its prognostic value in clinical practice

**DOI:** 10.1186/s12967-025-06402-9

**Published:** 2025-03-31

**Authors:** Jie Zhou

**Affiliations:** Shenzhen Hospital (Longgang), Beijing University of Chinese Medicine, Shenzhen, China

**Keywords:** Radiomics, High-grade glioma, IL7R, Prognosis

## Abstract

**Background:**

High-grade gliomas are among the most aggressive and deadly brain tumors, highlighting the critical need for improved prognostic markers and predictive models. Recent studies have identified the expression of IL7R as a significant risk factor that affects the prognosis of patients diagnosed with high-grade gliomas (HGG). This research focuses on investigating the prognostic significance of Interleukin 7 Receptor (IL7R) expression and aims to develop a noninvasive predictive model based on radiomics for HGG.

**Methods:**

We conducted an analysis using data from The Cancer Genome Atlas (TCGA) and The Cancer Imaging Archive (TCIA), focusing on a group of 310 patients diagnosed with high-grade gliomas. To evaluate prognosis, we applied both univariate and multivariate Cox regression analyses alongside Kaplan–Meier survival analysis. Radiomics features were extracted from specific regions of interest, which were outlined by two physicians using 3D Slicer software. For selecting the most relevant features, we utilized the Minimum Redundancy Maximum Relevance (mRMR) and Recursive Feature Elimination (RFE) algorithms. Following this, we developed and assessed Support Vector Machine (SVM) and Logistic Regression (LR) models, measuring their performance through various metrics such as accuracy, specificity, sensitivity, positive predictive value, calibration curves, the Hosmer–Lemeshow goodness-of-fit test, decision curve analysis (DCA), and Kaplan–Meier survival analysis.

**Results:**

The survival analysis encompassed a total of 310 patients diagnosed with high-grade glioma, sourced from the TCGA database. Patients were stratified into high and low expression groups based on the levels of IL7R expression. Kaplan–Meier survival curves and Cox regression analysis revealed that an increase in IL7R expression correlated with a decline in overall survival (OS). The median Intraclass Correlation Coefficient (ICC) for the assessed radiomic features was determined to be 0.869, with 93 features exhibiting an ICC of 0.75 or greater. Utilizing the mRMR and RFE methodologies led to the identification of a final set comprising eight features. The Support Vector Machine (SVM) model recorded an Area Under the Curve (AUC) value of 0.805, whereas the AUC derived from fivefold cross-validation was noted to be 0.768. Conversely, the Logistic Regression (LR) model produced an AUC of 0.85, with an internal fivefold cross-validation AUC of 0.779, indicating a more robust predictive capability. We developed Support Vector Machine (SVM) and Logistic Regression (LR) models, with the LR model demonstrating a more robust predictive capability. Further Kaplan–Meier analysis underscored a significant association between elevated risk scores from the LR model and OS malignancy, with a *P* value of less than 0.001. GSVA analysis showed the enrichment pathway of KEGG and Hallmark genes in the high RS group. Moreover, expression levels of the LOX gene and the infiltration of M0 macrophages were significantly heightened in the high-risk score group, alongside an increase in tumor mutation burden (TMB). Interestingly, the mutation frequencies of TP53 and PIK3CA were found to be lower in the high-risk score group when compared to their low-risk counterparts.

**Conclusion:**

IL7R expression is a vital prognostic marker in high-grade gliomas. The radiomics-based LR models demonstrate strong predictive capabilities for patient outcomes. Future investigations should aim to incorporate these insights into clinical practice to enhance personalized treatment approaches for patients with high-grade glioma.

**Supplementary Information:**

The online version contains supplementary material available at 10.1186/s12967-025-06402-9.

## Introduction

Glioma is the most common malignant cancer of the central nervous system, and its treatment is one of the most challenging problems in neuro-oncology [1, 2] Currently, the treatments for glioma include surgery, radiotherapy, chemotherapy and targeted therapy, etc. However, due to the high difficulty of its treatment, the rate of disability and death is also very high [3, 4]. The World Health Organization (WHO) classification of central nervous system tumours classifies [5] gliomas into grades I-IV, of which grades I-II are low-grade gliomas with low malignancy and relatively good prognosis, while grades III-IV are high-grade gliomas (HGG) with high malignancy and poor prognosis. Despite advances in surgical techniques, radiotherapy, and chemotherapy, the median survival for patients with GBM remains approximately 15 months, and the 5-year survival rate is less than 10% [6]. The classical prognostic indicators for gliomas, including clinicopathological features, Ki67, and other indicators, and CT, MRI and other imaging methods, can no longer meet the clinical needs of precision medicine [7]; new prognostic markers need to be further explored to stratify patients’ prognosis for individualized medical treatment and provide new indicators for individualized precision treatment.

The IL7R gene encodes a protein that is a subunit of the IL-7 receptor, a receptor protein on cell membranes. IL-7 is a member of the cytokine family with four antiparallel helices that bind type I cytokine receptors. It is produced by stromal cells and is required for lymphocyte development and homeostatic survival [8] IL-7 and IL-7R promote cell survival and inhibit apoptosis primarily through activation of the JAK, STAT5, and PI3K-AKT-mediated signalling pathways [9]. The IL7R gene plays a key role in the development and function of the immune system. The IL7R gene plays a key role in the development and function of the immune system, and mutations or abnormalities in the IL7R gene may be associated with the occurrence and development of a number of immune-related diseases, such as autoimmune diseases and immunodeficiency diseases [10, 11]. Recent studies have demonstrated the high efficacy of IL-7/IL-7 receptor (IL-7R)-based immunotherapy against various malignancies, both in animal models and in humans. In recent years, the progression-free survival and overall survival of glioma patients have been significantly increased by the introduction of genetically modified T-cells (CAR)-T cells expressing C7R and long-acting IL7 agonists [12]. Therefore, the study of the IL7R gene contributes to an in-depth understanding of the regulatory mechanisms of the immune system as well as the mechanisms of related diseases. In the context of HGGs, IL7R expression may play a role in tumor growth and immune evasion, making it a promising target for therapeutic intervention.

Currently, IL7R expression level can only be detected by invasive methods such as peripheral blood cytokine assays, fresh tissue-based mRNA or protein level assays, and paraffin tissue-based assays, all of which have significant limitations including high costs, operator dependency, and inability to reflect the tumor parenchyma accurately MR Imaging is the most accessible image data necessary for clinical diagnosis Artificial intelligence is gradually being applied to the imaging profession, causing a huge change in imaging. Radiomics data is a kind of high-throughput radiomic feature extraction, which can obtain a large number of image parameters, and is a non-invasive, dynamic detection and quantitative response to the characteristics of the tumour [13]. Radiomics has been widely used in clinical practice, and previous studies have shown that Radiomics can be used for early diagnosis and staging of HGG, as well as for assessing tumour heterogeneity and the microenvironment.

Based on the above factors, the present study innovatively proposed to non-invasively predict the mRNA expression of IL7R in HGG tissues by developing a radiomics-based predictive model, and assessed the correlation between the constructed radiomics model and the related genes and prognosis; at the same time, we also integrated bioinformatical analyses to explore the potential molecular mechanisms behind the expression of IL7R and its association with the immune microenvironment.

## Materials and methods

### Patients and datasets

Data regarding high-grade gliomas were sourced from the The Cancer Genome Atlas (TCGA, https://portal.gdc.cancer.gov/) and The Cancer Imaging Archive (TCIA, http://www.cancerimagingarchive.net/). This study concentrated on patients with pathologically confirmed grade III and IV gliomas who were receiving their first treatment, specifically focusing on primary solid tumors that had undergone RNA sequencing (RNA-seq). Inclusion criteria required initial diagnoses of grade III and IV gliomas, with a particular emphasis on primary solid tumors analyzed through RNA-seq. Additionally, the study mandated the availability of comprehensive clinical data and high-resolution MRI-enhanced imaging for each patient. Exclusion criteria were established to eliminate cases lacking complete clinical or survival information, specifically excluding individuals who survived for less than 1 month after their diagnosis. Additionally, samples characterized by inadequate quality of imaging were omitted from the analysis. A total of 310 patients with high-grade glioma in the TCGA database were included in the survival analysis. Number of samples, inclusion, and exclusion criteria for TCGA and TCIA data, add to the attached Table S1. Our research utilizes patient data from ethically sanctioned databases that are publicly accessible for research endeavors. Given the open-source nature of the data, our study is devoid of ethical dilemmas and potential conflicts of interest.

The R package ‘survminer’ (v0.4.9) https://github.com/kassambara/survminerwas employed, utilizing a cutoff value of cutoff = 0.5547 predicated on IL7R expression [14]. Transcriptomic sequencing data, along with clinical and follow-up information, were procured from the TCGA database, while medical imaging was acquired from the TCIA database.

### Calculation of KM curves, median survival times, and point-in-time survival rates

Kaplan–Meier survival curves were employed to illustrate variations in survival rates across different cohorts, with the median survival time denoting the duration corresponding to a survival rate of 50%. To assess the significance of survival rate differences among the groups, the log-rank test was utilized.

### Cox regression

Cox regression analyses were conducted utilizing the R packages “‘survival’ (v3.2.13) https://github.com/therneau/survival” and “forestplot’ (v2.0.1) https://github.com/LSYS/forestplot” [15]. The Cox proportional hazards model facilitates the examination of the relationship between one or several predictors and the incidence of survival outcomes. A univariate Cox regression approach was employed to evaluate the correlation of various factors impacting overall survival (OS), whereas a multifactorial Cox regression analysis was performed to ascertain the association of specific factors with survival outcomes. This methodology primarily aims to determine whether a given factor acts as an independent influence on OS and to investigate the roles of various contributing factors.

### Subgroup analyses and interaction tests

Univariate Cox regression was also utilized to carry out exploratory subgroup analyses, assessing the impact of IL7R expression (comparing high expression versus low expression groups) on the prognostic outcomes of patients across different covariate subgroups. The interaction between IL7R expression and other covariates was further evaluated using the likelihood ratio test.

### Radiomics

#### Image processing and outlining

**Fig. 1:**
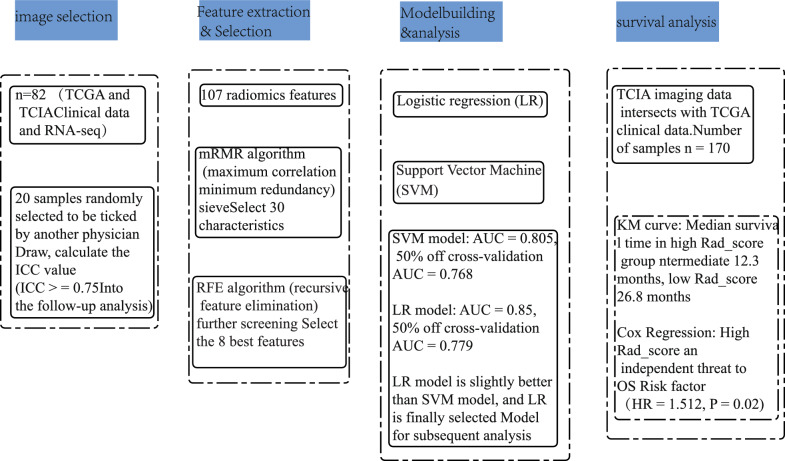
Image omics flow chart

The biometric data from The Cancer Genome Atlas (TCGA) were integrated with imaging data from The Cancer Imaging Archive (TCIA), yielding a sample size of *n* = 82. The data underwent normalization to derive radiomic features, comprising 107 features extracted via the pyradiomics’ (v3.0.1) https://github.com/AIM-Harvard/pyradiomics package. The entire tumor region was delineated manually using 3D Slicer software (version 4.10.2), focusing on gliomas as visualized in enhanced T1-weighted MRI scans, which included both enhanced and non-enhanced tumor areas [16]. Additionally, twenty samples were randomly selected for outlining by a separate physician. The complete workflow for the radiomics analysis is depicted in Fig. 1.

#### Intraclass correlation coefficient (ICC)

The intraclass correlation coefficient (ICC) was employed to assess the consistency of histological features extracted from volumes of interest (VOIs) based on the sketches provided by two physicians. After one physician completed the sketching of all cases, another physician randomly selected 20 samples through a “random number table method” for histological feature extraction. Generally, an ICC value of 0.75 or higher is indicative of excellent agreement, values ranging from 0.51 to 0.74 suggest moderate agreement, and values below 0.50 indicate poor agreement. Features exhibiting ICC values above 0.75 were subsequently filtered for further analysis using the R package “‘irr’ (v0.84.1) https://github.com/visinf/irr” [17].

#### Feature selection

The mRMR (Maximum Relevance Minimum Redundancy) algorithm was employed to identify 30 relevant features, followed by the application of the RFE (Recursive Feature Elimination) algorithm to refine the selection to the most optimal subset of features. The mRMR method assesses not only the dependence between each feature and the target variable but also the inter-correlation among the features themselves. This approach utilizes mutual information as a metric. In the context of mRMR, the relevance of a feature subset to a particular category is determined by averaging the information gain of individual features concerning that category. Conversely, redundancy among features is quantified by aggregating the mutual information values between pairs of features and normalizing this sum by the square of the total number of features in the subset.

RFE operates by ranking the predictors prior to model development and systematically removing those deemed less significant. This iterative process is designed to identify a specific group of predictors that can effectively enhance the model’s accuracy. The approach consists of repeatedly training the model, removing ‘*n*’ features that are deemed to have low importance after each round, and then reevaluating the significance of the features that remain. This cycle persists until the optimal subset of features is determined. Supplementary Documents1: Detailed criteria for selecting the eight radiomics features and data preprocessing steps, including normalization and standardization of radiomics features.

#### Imaging histological model selection and construction

The Support Vector Machine (SVM) algorithm utilizes support vectors to define high-dimensional hyperplanes that act as decision boundaries. In this study, we implemented the SVM algorithm using the “‘caret’ (v6.0.93) https://github.com/topepo/caret/” package in R to model the chosen image genomics features, which enabled us to predict gene expression levels effectively.

The logistic regression (LR) model, grounded in the principles of linear regression, employs a composite sigmoid function and is widely used for binary classification tasks., The formula is ${\varvec{g}}({\varvec{z}}) = {\varvec{y}}({\varvec{x}}) = \frac{{\mathbf{1}}}{{{\mathbf{1}} + {\varvec{e}}^{{ - {\varvec{\omega}}^{{\varvec{T}}} {\varvec{x}}}} }}$. In this study, the logistic regression algorithm was applied to analyze the histological features of filtered images. Using the “‘stats’ (v4.1.2) https://github.com/exelban/stats” package in R, we constructed a binary classification model designed to predict gene expression based on these features.

The evaluation of the models involved several metrics, such as accuracy (ACC), specificity (SPE), sensitivity (SEN), positive predictive value (PPV), and negative predictive value (NPV). To assess the calibration of the radiomics prediction model, calibration curves were generated, and the Hosmer–Lemeshow goodness-of-fit test was conducted. Additionally, the clinical utility of the radiomics prediction model was demonstrated through decision curve analysis (DCA).

### Clinical prognosis

#### Model predictions and GSVA enrichment analysis of high and low subgroups

The Radiomics score (RS) for the samples was derived from the radiomics model, subsequently categorizing the samples into dichotomous variables of Low and High. Expression matrices from 82 patients diagnosed with high-grade gliomas within The Cancer Genome Atlas (TCGA) were utilized to compute pathway enrichment scores for both KEGG pathway gene sets and Hallmark gene sets in each sample through Gene Set Variation Analysis (‘GSVA’ (v1.42.0) https://github.com/rcastelo/GSVA). A differential analysis was conducted employing the R package “‘limma’ (v3.50.0) https://github.com/cran/limma” to compare the RS high and low groups, with the top 30 pathways visualized using a threshold of |t|= 1. The threshold of |t|= 1 was chosen to identify the top 30 pathways based on effect size rather than statistical significance (*P* value or FDR). This approach was used to highlight pathways with the most substantial changes in expression, regardless of multiple testing corrections. Specifically, 186 pathways were analyzed within the KEGG pathway gene sets, and 50 pathways were assessed in the Hallmark gene sets enrichment analysis.

#### Differential analysis of gene expression linked to epithelial-mesenchymal transition

A total of 200 genes associated with the EPITHELIAL_MESENCHYMAL_TRANSITION pathway from the Hallmark gene set were compiled. The Wilcoxon test was employed to evaluate the expression differences of these 200 genes pertinent to epithelial-mesenchymal transition between the RS high and low groups, with statistical significance established at *P* < 0.05.

#### Differential analysis of immune cell abundance

Gene expression matrices from high-grade glioma patient samples were submitted to the CIBERSORTx database (https://cibersortx.stanford.edu/) to ascertain immune cell infiltration for each sample. For CIBERSORTx, no specific cut-off was applied; the tool provides relative proportions of immune cell infiltration. The disparities in immune cell infiltration levels between the RS high and low expression groups were analyzed using the Wilcoxon rank sum test.

#### Analysis of tumor mutational load (TMB)

Tumor mutational burden (TMB), defined as the quantity of somatic mutations per megabase of genomic sequence, serves as a potential predictive biomarker for identifying cancer patients who are likely to benefit from immune checkpoint inhibitors. Mutation data in MAF format were retrieved from the TCGA database (https://portal.gdc.cancer.gov/) for high-grade glioma samples. For TMB, the cut-off was determined based on the median value of mutations per megabase (mut/Mb) across the cohor. The TMB for these samples was computed using the maftools package, and differences in TMB between the RS high and low subgroups were assessed utilizing the Wilcoxon rank sum test.

#### Model prediction outcomes: analysis of RS mutations among high and low subgroups

Mutation information pertaining to patients with high-grade gliomas was retrieved from the TCGA Data Portal, yielding a sample size of 138 after intersecting with the radiomics dataset. The somatic variant data were formatted in Mutation Annotation Format (MAF) and subjected to analysis utilizing the R package ‘maftools’ (v2.10.0) https://github.com/PoisonAlien/maftools. The visualization focused on the top 15 genes exhibiting the highest mutation frequencies. The mutation data were processed through the R package ‘maftools’ (v2.10.0) https://github.com/PoisonAlien/maftools, and the visualization depicted the 15 genes with the most prevalent mutations.

## Results

### Patient demographics

In this study, we analyzed data from 310 patients diagnosed with high-grade gliomas sourced from the TCGA database. These individuals were stratified into two groups based on IL7R expression levels: the high-expression cohort (*n* = 100) and the low-expression cohort (*n* = 210), utilizing a median value of 0.5547 as the threshold for classification. The clinical characteristics of the subjects are detailed in. Notably, the age distribution revealed a statistically significant difference between the high- and low-expression groups of IL7R (*P* < 0.001).

### Survival analysis

**Fig. 2:**
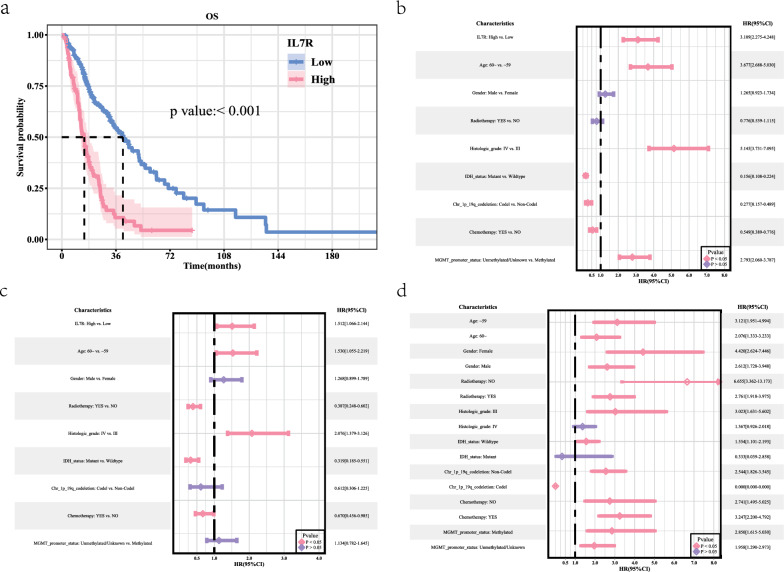
IIL7R is overexpressed in HGG patients and is associated with poor prognosis. **a** Kaplan–Meier curves were used to analyze the overall survival of the TCGA–TCIA cohort grouped by IL7R expression. Univariate (**a**) and multivariate (**b**) analyses of the TCGA–TCIA cohort. **c**, **d** Subgroup analysis and interaction test, univariate table and univariate forest plot

The study calculated the median survival durations and point-in-time survival rates, where the low-expression group exhibited a median survival time of 40.67 months, in contrast to 14.93 months for the high-expression group (Table 1). Kaplan–Meier survival curves indicated that elevated IL7R expression correlated with a decline in overall survival (OS), achieving a significant *P* value (*P* < 0.001) (Fig. 2a).

**Table 1:** Baseline data median survival time

	Records	n. max	n. start	Events	*rmean	*se (rmean)	Median	0.95 LCL	0.95 UCL
IL7R = Low	210	210	210	96	54.17524943	5.840429297	40.66666667	33.7	51.56666667
IL7R = High	100	100	100	76	25.55730114	5.525365444	14.93333333	12.06666667	17.9

### Cox regression analysis

Univariate analysis identified high IL7R expression as a statistically significant prognostic factor for OS (HR = 3.109, 95% CI 2.275–4.248, *P* < 0.001) (Fig. 2b). Further multifactorial analysis, after controlling for confounding variables, affirmed that high IL7R expression remained a significant risk factor for OS (HR = 1.512, 95% CI 1.066–2.144, *P* = 0.02) (Fig. 2c).

### Subgroup analyses and interaction tests

Subgroup analysis revealed that in patients aged 60 years or younger, elevated IL7R levels constituted a risk factor for OS (HR = 3.121, 95% CI 1.951–4.994, *P* < 0.001), demonstrating statistical significance. Similarly, for patients older than 60 years, increased IL7R expression was also a risk factor for OS (HR = 2.076, 95% CI 1.333–3.233, *P* = 0.001), which was statistically significant. The interaction test yielded a *P* value of 0.22, indicating no significant interaction between IL7R expression and the different age cohorts. Thus, it can be concluded that the influence of IL7R on OS was comparable across the two age subgroups (Fig. 2d).

## Radiomics

**Fig. 3:**
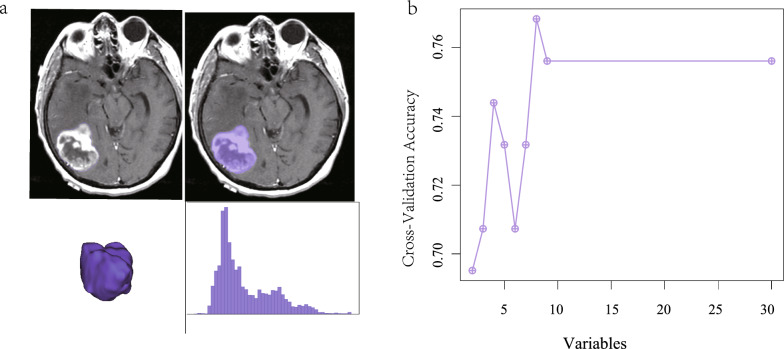
Picture processing. **a** Image sketching. **b** Feature selection

**Fig. 4:**
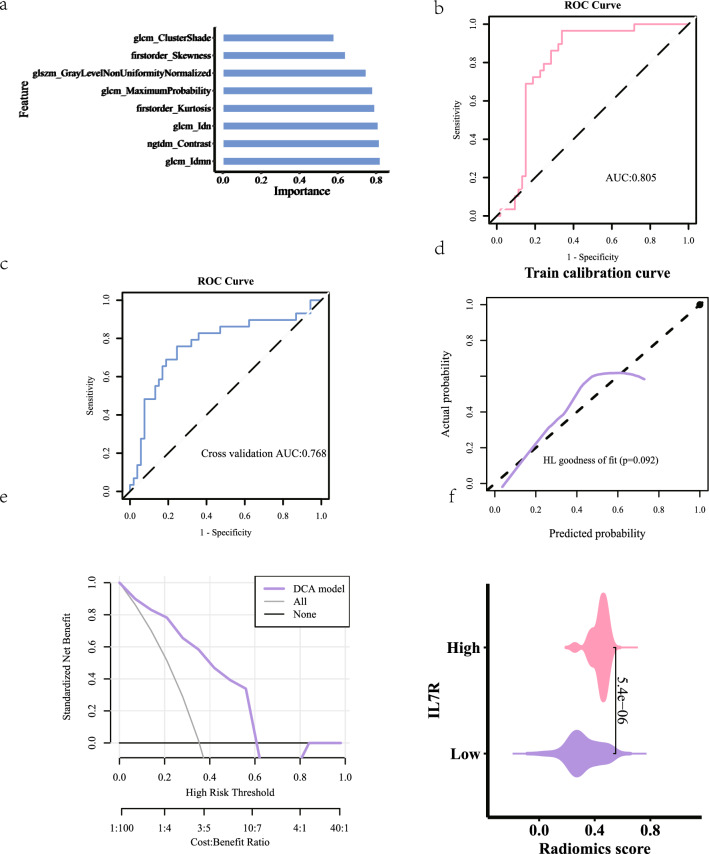
**a** Plot of feature importance degree in SVM model. **b** SVMModel AUC value. **c** Fivefold cross-validation AUC. **d** Calibration curves and HL goodness of fit. **e** DCACurve. **f** SVMAnalysis of differences between model groups

1. *Intraclass correlation coefficient (ICC)*: Two medical professionals delineated the Volume of Interest (VOI) and subsequently extracted radiomics features (refer to Fig. 3a). The median ICC for the imaging genomics features was calculated to be 0.869, with 93 features exhibiting an ICC value of 0.75 or greater, accounting for 86.9% of the total features (see Table 2). These features were included in the subsequent screening process. Further details can be found in the attached Table 2.
2. *Feature extraction and screening*: The minimum Redundancy Maximum Relevance (mRMR) algorithm was employed to eliminate extraneous features, resulting in a selection of 30 features. Subsequently, the Recursive Feature Elimination (RFE) algorithm was utilized to refine the selection to the most optimal features, ultimately identifying 8 significant features (Fig. 3b).
3. *Imaging group modeling and evaluation*: The two algorithms yielded differing importance rankings for the 8 features. In the Support Vector Machine (SVM) algorithm, the significance of the features is illustrated in Fig. 4a. The radiomics model demonstrated a robust predictive capability, as evidenced by the Receiver Operating Characteristic (ROC) curve, which indicated an Area Under the Curve (AUC) value of 0.805 (see Fig. 4b). The AUC for the fivefold cross-validation was determined to be 0.768 (Fig. 4c). The calibration curve and the Hosmer–Lemeshow goodness of fit test confirmed that the predicted probability of high gene expression by the radiomics model aligned well with the actual values (*P* > 0.05, see Fig. 4d). The Decision Curve Analysis (DCA) highlighted the model’s significant clinical utility (Fig. 4e). Furthermore, the comparative analysis of the SVM model revealed a statistically significant difference in the distribution of Rad_score between high and low gene expression groups (*P* < 0.001), with the group exhibiting elevated IL7R expression showing a higher Rad_score (Fig. 4f).

**Table 2:** Intraclass correlation efficient, ICC

	ICC ≥ 0.75	0.5 ≤ ICCC < 0.75	ICC < 0.5	ICC_Mean	ICC_Median
Percentage	0.869	0.103	0.028	0.9	0.97
Number	93	11	3	NA	NA

**Fig. 5:**
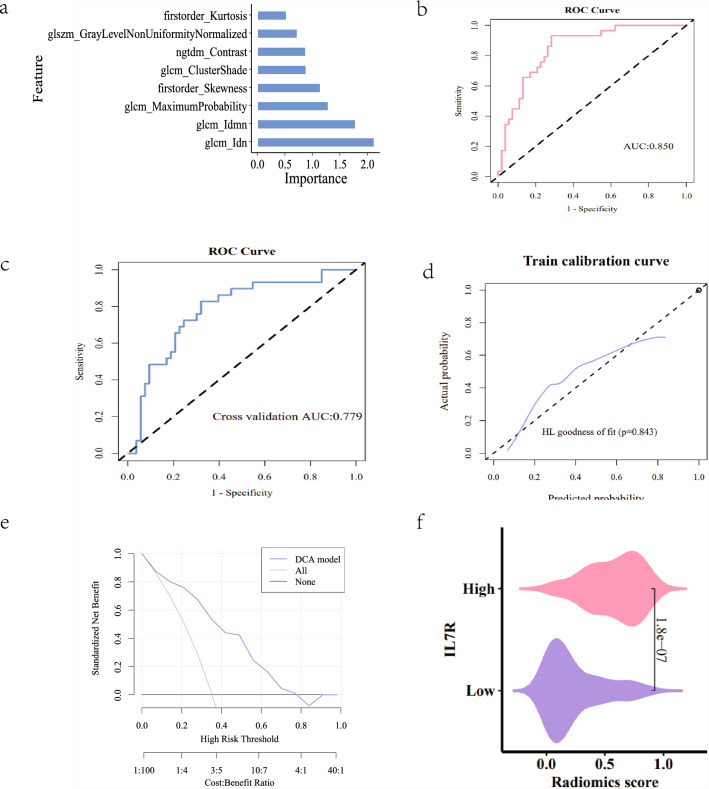
**a** Plot of feature importance degree in LR model. **b** LR Model AUC value. **c** Fivefold cross-validation AUC. **d** Calibration curves and HL goodness of fit. **e** DCACurve. **f** LR Analysis of differences between model groups

In the Logistic Regression (LR) model, the significance of the features is depicted in Fig. 5a. The results of the regression coefficients related to the features in the LR model are shown in Table 3. The radiomics formula is expressed as: Radiomics = Feature * Corresponding Coefficient (Estimate) + Intercept (Estimate) (refer to Table 3). The predictive efficacy of the radiomics model was confirmed by the ROC curve, revealing an AUC value of 0.85 (Fig. 5b). The AUC values for the fivefold internal cross-validation were calculated to be 0.779 (Fig. 5c). Similar to the SVM model, the calibration curve and Hosmer–Lemeshow goodness of fit test indicated that the predicted probability of high gene expression by the radiomics model was consistent with the actual values (*P* > 0.05, see Fig. 5d). The DCA also demonstrated the model’s clinical applicability (Fig. 5e). The comparative analysis between the LR model groups showed a significant difference in the distribution of Rad_score (*P* < 0.001), with the group showing high IL7R expression displaying a higher Rad_score (Fig. 5f).

**Table 3:** Regression coefficient of feature in LR model

	Estimate
(Intercept)	−1.017450318
original_ngtdm_Contrast	0.64299895
original_glcm_Idmn	3.787615188
original_firstorder_Kurtosis	−0.407920908
original_glcm_Idn	−5.01458914
original_glcm_MaximumProbability	0.698800646
original_glcm_ClusterShade	−0.35539405
original_firstorder_Skewness	0.881224081
original_glszm_GrayLevelNonUniformityNormalized	−0.441609203

The Delong test was employed to assess the Area Under the Curve (AUC) values for the models under consideration. The comparison of AUC values indicated no statistically significant difference between the Logistic Regression (LR) model’s AUC values in both the training set and the validation set (*P* = 0.073), suggesting an adequate model fit. Similarly, there was no significant difference observed in the AUC values of the Support Vector Machine (SVM) model between the training and validation sets (*P* = 0.885) (refer to Table 4), further supporting the conclusion of a well-fitted model. It is noted that the AUC value for the LR imaging genomics model marginally surpassed that of the SVM imaging genomics model. However, the Delong test confirmed that the differences between the two models were not statistically significant. Additionally, the AUC and other performance metrics of the LR imaging genomics model exhibited slight superiority over those of the SVM imaging genomics model; hence, the output value (Rad_score) from the LR imaging genomics model was selected for further clinical analysis.

**Table 4:** SVM, LR intermodel comparison

Train	Vali
0.073424714	0.885646837

*Model prediction*: The imaging data from The Cancer Imaging Archive (TCIA) were integrated with the clinical data from The Cancer Genome Atlas (TCGA), yielding a total of 170 intersecting samples. The Radiomics score for these samples was computed using the LR Radiomics Model. This score was subsequently combined with the clinical data, and the cutoff value for the Radiomics score was determined utilizing the survminer v0.4.9 package to categorize the data into Low/High dichotomous variables (RS). Based on the RS expression with a cutoff value of 0.449, patients were classified into a high expression group (*n* = 69) and a low expression group (*n* = 101). The clinical characteristics of these patients are detailed in Table 5. A statistically significant difference (*P* < 0.001) was observed in the distribution of tumor pathological grading and IDH status between the high and low RS expression groups.

**Table 5:** TCIA–TCGA pooled data

Variables	Total (*n* = 170)	Low (*n* = 101)	High (*n* = 69)	*P*
Age, *n* (%)				<0.001
~59	103 (61)	72 (71)	31 (45)	
60~	67 (39)	29 (29)	38 (55)	
Gender, *n* (%)				0.029
Female	75 (44)	52 (51)	23 (33)	
Male	95 (56)	49 (49)	46 (67)	
Radiotherapy, *n* (%)				0.681
No	26 (15)	14 (14)	12 (17)	
Yes	144 (85)	87 (86)	57 (83)	
Histologic_grade, *n* (%)				<0.001
III	54 (32)	50 (50)	4 (6)	
IV	116 (68)	51 (50)	65 (94)	
IDH_status, *n* (%)				<0.001
Wildtype	128 (75)	61 (60)	67 (97)	
Mutant	42 (25)	40 (40)	2 (3)	
Chr_1p_19q_codeletion, *n* (%)				0.002
Non-codel	155 (91)	86 (85)	69 (100)	
Codel	15 (9)	15 (15)	0 (0)	
Chemotherapy, *n* (%)				0.546
No	32 (19)	17 (17)	15 (22)	
Yes	138 (81)	84 (83)	54 (78)	
MGMT_promoter_status, *n* (%)				<0.001
Unmethylated/unknown	90 (53)	41 (41)	49 (71)	
Methylated	80 (47)	60 (59)	20 (29)	

**Fig. 6:**
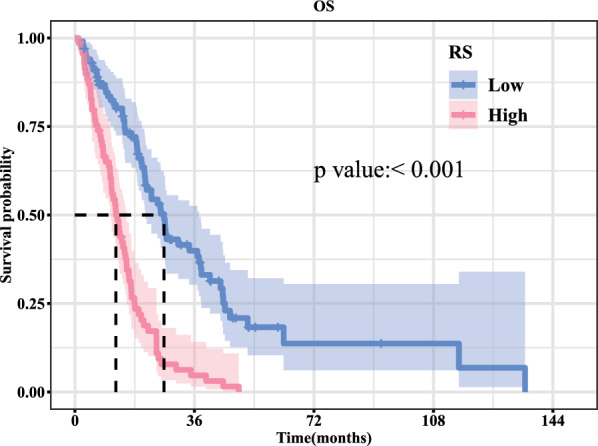
TCIA–TCGA Kaplan–Meier survival curves

*KM curve*: Kaplan–Meier survival curves were generated using the ‘survminer’ (v0.4.9) https://github.com/kassambara/survminerpackage in R to illustrate the survival rate variations among different groups for each variable. The median survival time, indicative of the duration corresponding to a survival rate of 50%, was found to be 12.3 months for the high RS group and 26.8 months for the low RS group (Table 6). The Kaplan–Meier curves demonstrated a significant association between high RS and reduced overall survival (OS) (*P* < 0.001), as depicted in Fig. 6.

**Table 6:** Median survival time of TCIA–TCGA pooled data

	Records	n. max	n. start	Events	*rmean	*se (rmean)	Median	0.95 LCL	0.95 UCL
RS = Low	101	101	101	64	39.62201795	5.142500211	26.8	21.26666667	37.36666667
RS = High	69	69	69	66	14.31145964	1.232942238	12.33333333	10.6	15.53333333

### Model predictions: RS GSVA enrichment analysis between high and low subgroups

**Fig. 7:**
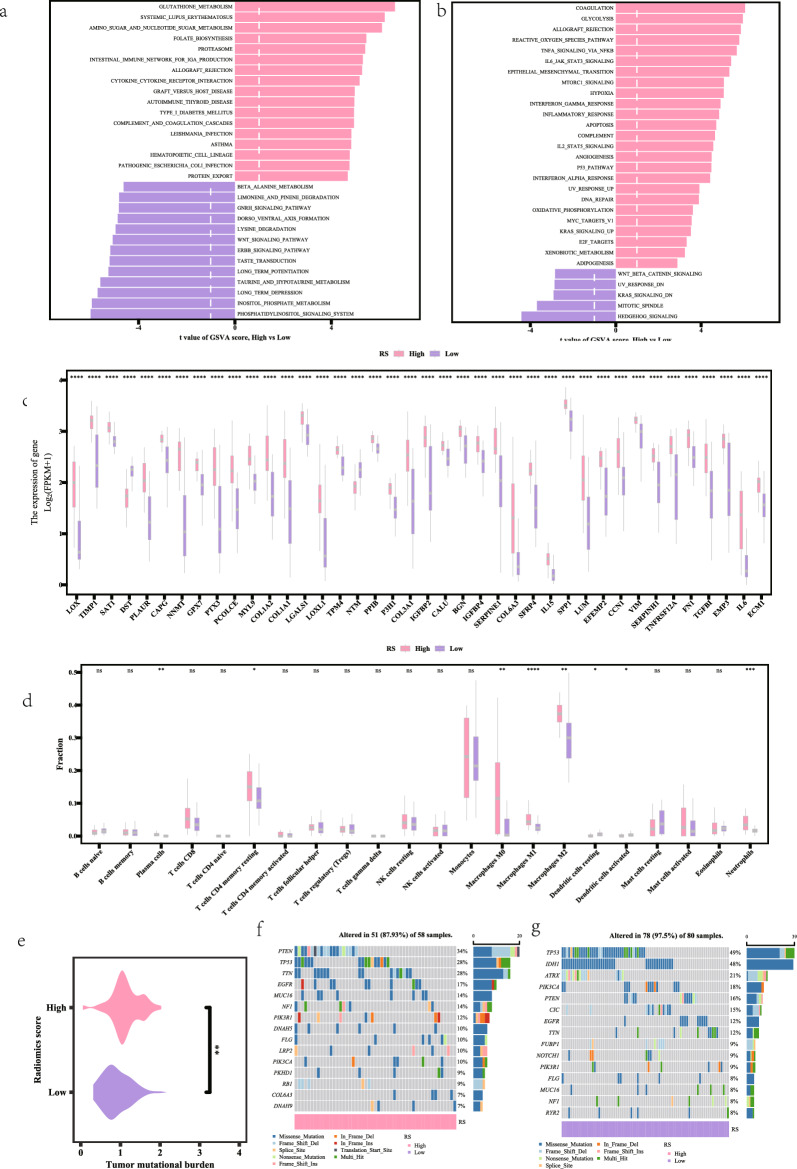
LR model prediction. GSVA enrichment analysis (**a**) was enriched in signaling pathways in KEGG gene set (**b**) is enriched in signaling pathways in the Hallmark gene set (**c**) and differential analysis of genes related to epithelial-mesenchymal transition (**d**). Analysis of the difference between immune cell abundance and immune cell abundance (**e**). Difference analysis with tumor mutation load (TMB). Gene mutation analysis (**f**). TP53gene mutation rate (**g**). TP53 and PIK3CA gene mutation rate. The annotation Multi_Hit indicates genes that are mutated multiple times in the same sample

We performed an analytical comparison of the GSVA results between high and low RS subgroups in patients with high-grade gliomas. The GSVA enrichment analysis revealed significant activation of cytokine—cytokine receptor interaction pathways in the high-risk group, suggesting a pro-inflammatory tumor microenvironment (Fig. 7a). Additionally, within the Hallmark gene set, the RS high subgroup displayed considerable enrichment in the L6_JAK_STAT3_SIGNALING pathway, as well as in signaling pathways associated with EPITHELIAL_MESENCHYMAL_TRANSITION (Fig. 7b).

### Differential analysis of gene expression associated with epithelial-mesenchymal transition

To assess the differences in gene expression pertinent to epithelial-mesenchymal transition between high and low RS groups, we employed the Wilcoxon test. The analysis revealed that the expression of the LOX gene was markedly elevated in the RS high expression group (*P* < 0.0001) (Fig. 7c). The differential EMT analysis highlighted the LOX gene’s role in promoting tumor invasiveness and metastasis.

### Differential analysis of immune cell abundance

We investigated the variations in immune cell infiltration between the RS high and low expression groups. Our results indicated that the infiltration of M0 macrophages was significantly higher in the RS high expression group (*P* < 0.01) (Fig. 7d). The immune cell abundance analysis showed increased M0 macrophage infiltration in the high-risk group, indicating a potential immunosuppressive environment.

### Tumor mutational load (TMB) distribution

The analysis of tumor mutational load (TMB) revealed a statistically significant difference between the RS high and low groups (*P* < 0.01), with the RS high group exhibiting elevated TMB values (Fig. 7e). TMB analysis revealed higher mutation burden in the high-risk group, which may correlate with increased genomic instability.

### Model prediction results: RS mutation analysis between high and low subgroups

Our mutation analysis indicated that Missense_Mutation was the predominant mutation type, followed by Nonsense_Mutation and Frame_Shift_Del. Notably, the mutation rate of the TP53 gene was observed to be equal to or greater than 20% in both the RS high and low subgroups. Furthermore, the mutation rates of the TP53 and PIK3CA genes were found to be lower in the RS high group compared to the RS low group (Fig. 7f, g).

The COX regression analysis of RS in conjunction with Kaplan–Meier (KM) survival analysis suggests that the classification of RS holds significant clinical prognostic relevance.

## Discussion

High-grade gliomas (HGGs) are a particularly aggressive type of brain tumor, known for their rapid growth and poor prognosis [18]. Despite notable progress in surgical techniques, radiation therapy, and chemotherapy, the overall survival rates for patients with HGG remain frustratingly low. The complexity of these tumors, characterized by significant molecular and cellular diversity, makes treatment challenging and highlights the urgent need for new diagnostic and prognostic biomarkers. The World Health Organization (WHO) classification system underscores the importance of molecular and genetic factors, such as IDH mutations and the co-deletion of chromosomes 1p/19q, which have been linked to patient outcomes [19]. Additionally, the tumor microenvironment and the immune response play crucial roles in tumor progression and the development of resistance to treatment, suggesting that a comprehensive approach is necessary to fully understand and effectively target these cancers [20, 21]. Among potential biomarkers, the interleukin-7 receptor (IL7R) has gained attention for its role in immune regulation and tumor progression, indicating its promise in the ongoing search for effective treatment strategies.

Gaining insights into the expression patterns of IL7R and its influence on patient prognosis could help develop more personalized therapeutic strategies. The variability in IL7R’s molecular expression levels, along with the lack of validated assessment tools, has led researchers to explore imaging modalities. Among these, radiomics shows promise in predicting the mutational status of IL7R molecules, allowing for non-invasive evaluations that can guide individualized clinical decision-making.

In this study, we established a radiomics model leveraging enhanced MRI imaging through machine learning algorithms to non-invasively predict IL7R mRNA expression in HGG tissues. We integrated transcriptomic data sourced from the TCGA database alongside imaging data from the TCIA database to examine the correlation between the radiomics model, gene expression, and clinical outcomes [22]. Our analysis included survival data from 310 HGG patients, categorized by IL7R expression levels. The findings from both univariate and multivariate Cox regression analyses indicated a significant association between IL7R expression and patient prognosis. Kaplan–Meier survival analysis further corroborated that the median survival time for patients with high IL7R expression was 14.93 months, in contrast to 40.67 months for those in the low-expression group, suggesting that elevated IL7R levels correlate with diminished overall survival. This indicates the potential of IL7R as a prognostic marker. Additional bioinformatics analyses were conducted to investigate the underlying molecular mechanisms and the relationship between IL7R expression and the immune microenvironment, thereby providing a comprehensive understanding of these interactions [23].

This study represents a significant advancement in the non-invasive prediction of interleukin-7 receptor (IL7R) mRNA expression in high-grade gliomas (HGG) by enhanced magnetic resonance imaging (MRI)-based radiomics models [24]. By integrating machine learning algorithms, particularly support vector machine (SVM) and logistic regression (LR) models, we developed a powerful prediction framework. Elevated IL7R expression was associated with decreased overall survival (OS), confirming the existing literature linking IL7R to immune responses, particularly in the context of tumor biology and T cell dynamics [25]. Previous studies have focused on the histopathological and molecular features of HGG [26, 27]. This study not only confirms the role of IL7R as a prognostic marker, but also integrates radiomics to enhance predictive modeling, filling a critical knowledge gap. It was demonstrated by Kaplan–Meier (KM) analysis and Cox regression modelling that high IL7R expression was significantly associated with poorer overall survival (OS) in patients with HGG. Immunotherapy with IL-7/IL-7 receptor (IL-7R) has high efficacy in a wide range of malignant tumours and has been demonstrated in animal models and in humans. Pathophysiological role of IL-7R in gliomas that survival of glioma patients starts to improve significantly after immunotherapy [28].

Our research distinguishes itself by integrating radiomics with bioinformatics to forecast IL7R expression and its clinical implications. Furthermore, this study represents the inaugural effort to authenticate these results employing non-invasive imaging methodologies. This pioneering strategy not only augments our comprehension of the molecular underpinnings of high-grade gliomas (HGG) but also offers a pragmatic tool for clinical decision-making.

By employing advanced radiological techniques alongside sophisticated feature selection methods, specifically Minimum Redundancy Maximum Relevance (mRMR) and Recursive Feature Elimination (RFE), we successfully optimized a selection of radiological features to pinpoint the eight most significant ones for building our predictive model. These selected features were then utilized to create prediction models using Support Vector Machine (SVM) and Logistic Regression (LR). Previous studies have shown that machine learning techniques, including SVM and logistic regression, can greatly improve prognostic accuracy [29]. Our models were rigorously assessed using various metrics such as accuracy (ACC), specificity (SPE), sensitivity (SEN), and positive predictive value. Notably, the logistic regression model demonstrated impressive performance, achieving an area under the curve (AUC) of 0.85, indicating its potential for application in clinical settings; consequently, we decided to adopt the LR model for further use.

Our study not only clarifies the prognostic importance of IL7R but also highlights significant biological pathways associated with increased IL7R expression, such as the cytokine-cytokine receptor interaction and the IL6-JAK-STAT3 signaling pathways. These findings are consistent with earlier research that demonstrates IL7R’s influence on immune responses and the tumor microenvironment [30]. Notably, the elevated expression of the LOX gene, along with a higher presence of M0 macrophages in the high-risk score group, supports the idea that IL7R may contribute to creating an immunosuppressive environment in gliomas. IL7R signaling contributes to immune evasion by promoting M0 macrophage polarization toward an immunosuppressive M2 phenotype, suppressing cytotoxic T—cell activity, and enhancing tumor cell survival through JAK—STAT3 pathway activation. These mechanisms collectively foster an immunosuppressive niche that facilitates tumor growth and therapy resistance. As a result, IL7R stands out as both a prognostic marker and a potential target for therapy. This underscores the need for further investigation into the therapeutic modulation of IL7R and its related pathways to enhance treatment outcomes for patients suffering from high-grade gliomas.

The prognostic significance of IL7R expression in high-grade gliomas is supported by its association with reduced overall survival and its role in promoting tumor aggressiveness through immune evasion and microenvironment remodeling.

Despite the encouraging results, it is crucial to acknowledge certain limitations in our study. Although we had a considerable sample size of 82 individuals sourced from the TCGA and TCIA databases, this cross-sectional group may limit the generalizability of our findings. Furthermore, the retrospective nature of the study and reliance on publicly available datasets could introduce selection bias. Using a single threshold for IL7R expression (0.5547) might not fully capture the range of expression levels and their clinical implications. Future research should aim to validate our results in larger, multicenter prospective cohorts and explore the varying spectrum of IL7R expression more thoroughly. Additionally, while our radiomics model showed improved predictive accuracy, it requires further refinement and validation in diverse clinical settings to confirm its reliability and practical application in routine clinical practice.

## Conclusion

In conclusion, our study underscores the promising role of radiomics in conjunction with machine learning algorithms for predicting IL7R expression in high-grade gliomas (HGG) and its implications for prognosis. The results reveal a notable link between elevated IL7R expression and reduced overall survival rates, indicating that IL7R may serve as an important biomarker for patient stratification and tailored treatment approaches. Nevertheless, to confirm these findings and enhance predictive models, future research should involve larger prospective cohorts and multi-institutional data. Furthermore, incorporating additional molecular and clinical factors could strengthen the reliability and clinical applicability of radiomics-based methods, ultimately aiding in the better management and prognosis of patients with high-grade gliomas. MRI radiomics models have the potential to non-invasively predict IL7R levels in high-grade gliomas, which are closely associated with clinical outcomes.

## Supplementary Information

**Additional file 1.**

**Additional file 2.**

**Additional file 3.**

**Additional file 4.**

## Data Availability

The data supporting the findings of this study are available from the corresponding author upon reasonable request.

## Abbreviations

HGG: High-grade gliomas
SVM: Support vector machine
LR: Logistic regression l
