# Supplementary material for: Construction of enhanced MRI-based radiomics models using machine learning algorithms for non-invasive prediction of IL7R expression in high-grade gliomas and its prognostic value in clinical practice
Source: J Transl Med. 2025 Mar 31;23:383. doi: 10.1186/s12967-025-06402-9 (PMC11959755; doi:10.1186/s12967-025-06402-9)
Supplement: Supplementary file 1 — Additional file 1. [file 12967_2025_6402_MOESM1_ESM.docx]

**TCGA** **_** **GBM** **and** **TCGA** **_** **LGG** **clinical** **data** **Get** **rid** **of** **Number** **of** **remaining** **samples**

| Total number of cases | - | 1114 |
| --- | --- | --- |
| Select the patients for the first treatment | 42 | 1072 |
| Gliomas with pathological grade G3 and G4 were selected | 310 | 762 |
| Those with missing survival data and a survival time shorter than one month were excluded | 43 | 719 |
| Missing clinical data were excluded | 127 | 592 |
| Primary solid tumors with RNA-seq data were available | 282 | 310 |
| **TCGA** **_** **GBM** **and** **TCGA** **_** **LGG** **to** **enhance** **T** **1** **WI** **Get** | **rid** **of** | **Number** **of** **remaining** **samples** |
| Total number of cases | - | 458 |
| Evaluation review (excluding poor image quality), samples with high-grade gliomas were selected | 284 | 174 |
| Samples with clinical data and RNA-seq data were selected | 92 | 82 |
| Samples with available clinical data were screened out | 4 | 170 |
